# Between hunter and climate: the effects of hunting and environmental change on fecal glucocorticoid metabolite levels in two sympatric ungulate species in the Ruaha–Rungwa ecosystem, Tanzania

**DOI:** 10.1093/conphys/coad002

**Published:** 2023-02-07

**Authors:** Kwaslema Malle Hariohay, Louis Hunninck, Peter S Ranke, Robert D Fyumagwa, Rupert Palme, Eivin Røskaft

**Affiliations:** Department of Wildlife Management, College of African Wildlife Management, Mweka, P. O. Box 3031, Moshi, Tanzania; Department of Biology, Norwegian University of Science and Technology, Realfagbygget, NO-7491, Trondheim, Norway; Department of Biology, Norwegian University of Science and Technology, Realfagbygget, NO-7491, Trondheim, Norway; Department of Natural Resources and Environmental Sciences, University of Illinois Urbana-Champaign, Urbana, IL 61801, USA; Department of Biology, Norwegian University of Science and Technology, Realfagbygget, NO-7491, Trondheim, Norway; Center for Biodiversity Dynamics (CBD), Department of Biology, Norwegian University of Science and Technology, Realfagbygget, NO-7491, Trondheim, Norway; Department of Wildlife Management, Tanzania Wildlife Research Institute (TAWIRI), P.O. Box 661, Arusha; Department of Biomedical Sciences, University of Veterinary Medicine, Vienna; Department of Biology, Norwegian University of Science and Technology, Realfagbygget, NO-7491, Trondheim, Norway

**Keywords:** trophy hunting, stress, population decline, fecal glucocorticoid metabolites, Anthropogenic activities

## Abstract

Understanding the drivers of animal population decline is a key focus of conservation biologists. Anthropogenic activities such as hunting have long been established as potentially detrimental to a population’s persistence. However, environmental perturbations such as increased temperature variability, exacerbated by climate change, can also have important effects on animal populations. Animals can respond to these challenges by adjusting both their behavior and physiology. We measured fecal glucocorticoid metabolites (FGMs) of common impala (*Aepyceros melampus*) and greater kudu (*Tragelaphus strepsiceros*), both currently in stable populations, to examine effects of hunting, forage availability, daily variability in temperature and group size on their physiological stress response. The study was conducted across two adjacent protected areas, (i) one non-hunted area (Ruaha National Park; RNP) and (ii) one area used for trophy hunting (Rungwa Game Reserve; RGR). Both impala and kudu had significantly higher FGM levels in the area that allows hunting, while FGM levels decreased with increasing forage availability and increasing daily temperature. Moreover, impala (but not kudu) had lower FGM levels with larger group sizes. Our results indicate that the management regime can significantly alter the physiological state of wild ungulate populations. We also highlight the importance of considering the combined effects of anthropogenic, environmental and social contexts when studying the stress response of wild populations. Our results emphasize the value of protected areas and continued monitoring of hunting quota in order to maintain ungulate populations that are less vulnerable to population declines.

## Teaser

We studied how anthropogenic and environmental disturbances affect fecal glucocorticoid metabolite (FGM) levels of impala and kudu. Both species had higher FGM levels when subjected to hunting, while FGM levels decreased with increasing forage availability. Our results indicate that management regimes can affect the physiological state of wild ungulate populations.

## Introduction

Anthropogenic activities such as tourism and hunting have long been established as important sources of disturbance to animal populations (Bateman and Fleming, 2017). However, environmental perturbations, including increased daily and seasonal temperature variation, can also have important negative effects on animal populations, and may be exacerbated by climate change (Niang *et al.,* 2014). Animals respond to such challenges through various behavioral and physiological mechanisms (Sih *et al.,* 2011). These physiological changes have become an important tool to monitor wild populations (Madliger *et al.,* 2018). However, few studies have studied the interactive effects of both anthropogenic activities and environmental perturbations on animal physiology (Santicchia *et al.,* 2020), and fewer studies still have investigated between-species differences in this response to similar disturbances (Pirotta *et al.,* 2018; Gairin *et al.,* 2022; Seebacher, 2022). Here, we investigate how the physiological state of two common antelope species are affected by both human activities, seasonal changes in food availability and temperature, and social context.

Protected areas, including national parks and game reserves, are increasingly impacted by encroachment from human activities such as crop farming (Hariohay *et al.,* 2020), logging for timber, hunting for bushmeat and livestock incursions (Knappa *et al.,* 2017; Kyando *et al.,* 2017; Hariohay *et al.,* 2019). Legal trophy hunting too has increased in game reserves (Hariohay *et al.,* 2018). These human activities can have direct and indirect effects on populations (Frid and Dill, 2002). For example, fewer calves were observed in Rungwa Game Reserve (RGR), an area that allows trophy hunting, compared to Ruaha National Park (RNP), a strictly protected area (Hariohay *et al.,* 2018). Similarly, impala and greater kudu populations were more vigilant and were more female skewed in partially protected areas compared to strictly protected areas (Hariohay *et al.,* 2018; Setsaas *et al.,* 2018). Greater kudu population had, on average, more vigilant individuals per group and higher flight initiation distance in RGR (trophy hunting area) as compared to populations in RNP (non-hunted area; Hariohay *et al.,* 2018). However, even relatively recent changes in management regimes can have significant effect on populations; impala population densities and flight behavior were shown to considerably improve after 15 years of stricter conservation management in the Serengeti ecosystem (Flølo *et al.,* 2021).

Regulated hunting is a common population management tool, which can promote plant regeneration and ecosystem health (Lindsey *et al.,* 2006; Di Minin *et al.,* 2016). The ‘2007 Wildlife Policy of Tanzania’ provides for a variety of wildlife utilization forms within the country (URT, 2007). The list is broad, comprising of game viewing, tourist hunting, farming, breeding, ranching, eco-tourism, zoos and game sanctuaries. Regardless of the utilization form or actor, the main purpose is to ensure that wildlife contributes adequately to socio-economic development without adversely impairing their conservation. The wider options for wildlife utilization apart, the tourism industry in the country continues to be dominated by game viewing and trophy hunting. Game viewing is non-consumptive and generally considered best practice in wildlife-rich ecosystems with good accessibility and visibility, but demands substantial investment in visitor amenities. In contrast, trophy hunting, while regulated, is a consumptive undertaking and is often carried out in extremely remote and rugged wildlife refuges with dense vegetation and poor wildlife numbers (Mahoney and Geist, 2019). Due to their remoteness, trophy hunting areas also tend to have facilities and infrastructure that are not to the satisfaction of a typical game viewer. Both management types therefore apply to different areas and can both help protect natural areas and wildlife populations (Lindsey *et al.,* 2006).

Antelope populations are largely regulated by forage availability (Hopcraft *et al.,* 2010). The seasonal changes in forage availability in East-African savannas can be substantial and, with climate change, are poised to become larger and more severe (Codron *et al.,* 2007; Midgley and Bond, 2015). In this study, we used the normalized difference vegetation index (NDVI; NASA MODIS; Didan, 2015) as a proxy for forage availability. NDVI measures primary productivity of a surface, based on the amount of near-infrared (NIR) and red light that is reflected; chlorophyll in green vegetation strongly reflects NIR while it mostly absorbs red light frequencies (Didan, 2015). This proxy is therefore useful to quantify the forage availability (e.g. nutritional sprouting grasses) over a large spatial and temporal extent (Pettorelli *et al.,* 2005). However, several caveats need to be considered when using NDVI data, including cloud cover, plant community and topography, as these factors can affect NDVI estimates (Pettorelli *et al.,* 2011; Pettorelli *et al.,* 2012). NDVI presents a powerful method for ecologists to relate large-scale vegetation changes to changes in animal physiology and behavior, and several studies have previously applied this remote sensed proxy in African savanna ecosystems (Stabach *et al.,* 2015; Hunninck *et al.,* 2020b).

Animal stress is defined as an environmental stimulus that causes an imbalance in homeostasis of an organism (Boonstra, 2013). External disturbances can trigger the release of hormones into the blood, resulting in an increased energy mobilization and facilitating an animal’s fight-or-flight response (Tingvold, 2011). The resultant change in behavior or physiology of the animal is known as the stress response (Tingvold *et al.,* 2013; Giudice *et al.,* 2018; Madliger *et al.,* 2018). These stress responses can be energetically costly, and any physiological or behavioral mechanism to mitigate stressors is at the cost of diverting energy from other physiological functions (Sapolsky *et al.,* 2000; Deng *et al.,* 2018; van de Ven *et al.,* 2019; Pérez-Barbería *et al.,* 2020). Although the stress response is an adaptive mechanism, being beneficial for how animals cope with challenges and thereby increase their overall fitness (Boonstra, 2013), chronic stressors—when stressors are often recurring or long in duration—resulting in sustained increases in glucocorticoid (GC) levels, may increase pathology of the animal (Sapolsky *et al.,* 2000; Sheriff *et al.,* 2011; Vilela *et al.,* 2020). Therefore, when exposed to a chronic stressor, the resultant elevated GC levels can indicate that the animal experiences an imbalance to its homeostasis (Harris, 2015). Stress in animals can be measured in a multitude of ways, including behavioral observations (Nyahongo, 2008; Hariohay *et al.,* 2018), bio-telemetric methods (Heylen and Nachtsheim, 2018) and measuring fecal glucocorticoid metabolites (FGMs; (Palme, 2019). Measuring FGMs is a non-invasive, quick, cheap and now routinely used method to assess adrenocortical activity. FGMs provide an integrated measure of adrenocortical activity over a certain time, depending upon the species (up to several hours in ruminants). Unlike plasma GC, FGM levels are less sensitive to minor fluctuations and a better proxy for chronic stress (Palme *et al.,* 2005; Hamilton *et al.,* 2011; Sheriff *et al.,* 2011; Tingvold *et al.,* 2013; Dantzer *et al.,* 2016).

Anthropogenic activities such as hunting and tourism are increasingly pervasive stressors as human–wildlife interactions continue to increase (Lunde *et al.,* 2016; Madliger *et al.,* 2018). Several studies have shown that FGM concentrations are higher in populations that experience human interactions, are hunted or reside in partially or non-protected areas, for example, African elephants (Ahlering *et al.,* 2013; Hunninck *et al.,* 2017) and mountain hares (*Lepus timidus*; (Rehnus *et al.,* 2014). Similarly, studies show that when animals faced environmental challenges, such as prolonged decreases in forage availability, FGM concentrations increased sharply (Stabach *et al.,* 2015; Hunninck *et al.,* 2017). Conversely, when high quality forage was widely available, FGM levels were found to be relatively low (Hunninck *et al.,* 2020a). FGM concentrations have also been shown to respond to fluctuations in temperature, where higher FGM—resulting in a higher mobilization of energy required for physiological thermoregulation—let animals cope with colder conditions (Huber *et al.,* 2003; Dalmau *et al.,* 2007; Santos *et al.,* 2018). Beside anthropogenic and environmental sources of disturbance, the social context of an animal can have considerable effects on an animal’s stress response (Creel *et al.,* 2013). For territorial animals such as the impala (Jarman and Jarman, 1973), territorial males were shown to have higher FGM levels compared to their non-territorial counterparts (Hunninck *et al.,* 2020c). However, for females, larger group sizes could have a buffering effect, decreasing the stress response (Hennessy *et al.,* 2009). Larger group sizes would result in lower individual vigilance, that is, many-eyes hypothesis (Pulliam, 1973). Furthermore, larger group sizes may lead to a decreased individual predation risk through dilution or confusion effects, further decreasing the stress response (Hamilton, 1971; Roberts, 1996; Goodale *et al.,* 2019). However, animals will choose to live in large groups only if the benefits (e.g. avoiding predation) outweigh the costs (e.g. foraging competition). Considering the various sources of disturbance is pivotal to fully understand how an animal responds to external stressors. However, although different species can vary greatly in how they cope with different perturbations, little is known about how different sympatric species respond physiologically to such stressors.

Our aim was to assess the effects of trophy hunting and environmental and social context on FGM levels in two large ungulate species in the Ruaha–Rungwa ecosystem, Tanzania. We hypothesized that the animals in the RGR, where trophy hunting is legally conducted, would have higher levels of FGMs compared to animals in the non-hunted RNP, where only non-consumptive tourism is conducted. Secondly, we hypothesized that FGM levels would decrease with increasing forage availability, as food is abundant. Thirdly, we hypothesized that with decreasing temperatures, FGM levels would increase to facilitate thermoregulation. Lastly, we hypothesized that with larger in group sizes, the stress response would be buffered, and thus, FGM levels would decrease.

## Methods

### Study area

**Figure 1:**
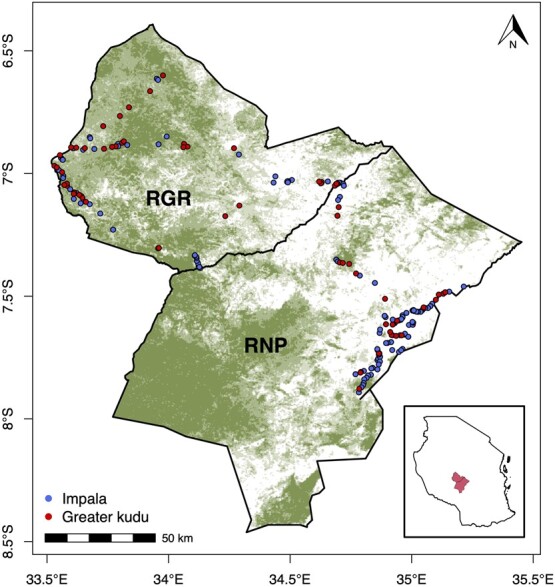
Location of impala (blue) and kudu (red) fecal samples that were collected in RGR and RNP. The inset shows the location of the study area in central Tanzania. The base map shows the percent woody cover, with darker green cells indicating a high relative percent woody cover and white areas indicating no woody cover; across the study system, the median woody cover was 7% (range: 0–59%).

The study was conducted across two management regimes, RGR and RNP, situated in south-central Tanzania (Fig. 1). RNP together with the surrounding game reserves (including RGR) constitutes the large Ruaha–Rungwa ecosystem covering a continuous area of ~45 000 km^2^. Elevation ranges from 800 to 1800 m, and the area receives an average amount of annual rainfall of 873 mm with a single wet season occurring from November to May and with the highest rainfall levels recorded in December and January. Temperatures range from an average of 21.5°C from June–July to 26.5°C from August–October (Marttila, 2011).

### Study species

Impala (*Aepyceros melampus*) is a medium-sized, sexually dimorphic antelope, common in eastern Africa (Averbeck, 2002). Impala inhabit savanna grasslands and woodlands situated close to water sources and are considered water dependent; impala have a relatively small home range (Jarman and Jarman, 1973). They are mixed foragers of grasses, forbs, monocots, dicots and foliage, and travel between habitats between seasons due to variability in food availability (Wronski, 2002; Marshal *et al.,* 2012). They live in three distinct social groups: (i) female herds with a territorial male, (ii) bachelor herds (males of different ages) and (iii) single territorial males. In this study, a group was defined by individuals behaving in a coordinated manner either moving together in same direction or engaged in the same activity at any one time, within a distance of less than a hundred meters.

Greater kudus (*Tragelaphus strepsiceros*) are found in woodlands, as they are browsers that eat leaves and shoots. In the dry season, they eat fruits for their liquid content and for the natural sugars that they provide (de Garine-Wichatitsky *et al.,* 2004). While they are not in groups with females, male kudus can be found in bachelor groups or, more likely, solitary (Kie, 1999; Hoffmann, 2016). Males are seen with females only during the mating season when they form groups of generally 5–15 kudus, including offspring (Hoffmann, 2016).

Both species are common in the Ruaha–Rungwa ecosystem and have a similar ecology, and were therefore chosen as focal species for this study.

### Fecal sample collection

A total of 312 fecal samples were collected in both areas across two years, in 2016 from 13 to 30 October, and in 2017 from 24 August to 8 September, for a total of 32 days. Following Millspaugh and Washburn (2004), we collected fecal samples rapidly (here, within 25 minutes) after defecation to avoid environmental degradation of FGMs. When an animal defecated, we identified the age and sex of the animal, measured the distance to the animal and took a photo. A person then walked to the place of defecation, and this distance was measured to make sure it was the exact same location as the studied animal had occupied. This procedure made it possible to accurately obtain the sex and age for each animal from which a fecal sample was obtained and importantly. After collection we placed the sample(s) on ice in a cooler box. Pellets from the whole defecation were collected to minimize individual variation, and an average of 1.5 (range, 1–11) animals was sampled from each group. Only adult females were sampled, and samples contaminated by urine were not collected as the hormone metabolites in urine can bias FGM results (Sheriff *et al.,* 2011; Madliger *et al.,* 2018). We collected fecal samples from 232 impala and 80 greater kudus.

### Lab processing

Samples were kept in a container with absolute alcohol and placed on ice in a cooler box throughout the fieldwork and placed in a −20°C freezer daily. At the end of each field season, the samples were transported by car (2 days; kept cool in a −20°C freezer) placed in a freezer at Serengeti Research Center. All samples were processed within two months after collection. For lab processing, we thawed the samples and then mixed pellets from each sample by hand to account for within-sample variation that can bias assay results (Millspaugh and Washburn, 2003; Vilela *et al.,* 2020). We then transferred 0.5 g of mixed, wet feces and 5-ml 80% ethanol to a centrifuge tube (Nunc®, 10 ml), then we homogenized the mix (Omni μH) and centrifuged the samples for 20 minutes at 1200 g (Unico Powerspin ™ LX). Then we transferred 1-ml supernatant from each sample to a 2-ml microtube, and let the samples dry at room temperature. FGMs were measured with an 11-oxoetiocholanolone EIA, first described by Möstl *et al.* (2002), which measures metabolites with a 5β-3α-ol-11-one structure. This EIA has been specifically validated for impala (Chizzola *et al.,* 2018), but not for Greater kudu. However, this particular EIA has been successfully validated for every ruminant species tested so far (Palme, 2019) and is therefore very likely to accurately measure FGM concentrations in Greater kudu as well.

### Land surface temperature

Because there is no weather station in the study area, and therefore no direct temperature data available, we used a previously validated technique to estimate daily ambient temperature by collecting data on land surface temperature (LST; Hunninck *et al.,* 2020a). We extracted the average daily LST estimate experienced by the sampled animal on the day and location of sampling (MOD11A1 MODIS/Terra; Wan *et al.,* 2015). However, because the samples in 2016 were collected in late October in 2016 compared to early September in 2017 (Fig. 4A), average temperature experienced across all individuals were significantly higher in 2016 (*F*_1, 230_ = 33.44, *P* < 0.001; Fig. 4B). LST values experienced by sampled individuals in 2016 averaged 42.3°C (SE = 0.39), while in 2017 39.0°C (SE = 0.41; Fig. 4B). While this proxy correlates strongly with ambient temperature, LST values tend to be higher than ambient temperatures.

### Statistical methods

Impala and kudu were analyzed separately but with identical methods. We assessed variation in FGM levels using linear mixed-effects models in ‘*lme4*’ (Bates et al. 2015) using R (R Core Team, 2020). This method allowed us to estimate the effect of one predictor, while controlling for the other predictors included in the model. Models were fitted using log-transformed FGM levels as the response to ensure normal distribution of model residuals. Main fixed predictors included management regime (i.e. non-hunting in RNP versus hunting in RGR), NDVI, LST (we included LST as a quadratic effect, as FGMs could be elevated at both very low and very high temperatures) and group size (impala: median = 6, range: 1–120; kudu: median = 5, range: 1–125). We compared five models for both impala and kudu, as we were interested in whether management regime interacted with the effect of the environmental variables on FGM levels. These models were a null model, a no-interaction model and three models with a single interaction between management regime and each environmental variables (NDVI, LST and group size). We used Akaike’s Information Criterion adjusted for small samples sizes (AICc; Burnham and Anderson, 2002) to assess which of the five models best fit our data. Models were considered significantly better fit to the data if ∆AICc > 2. We accounted for non-independence within groups of animals by adding the group identity as a random factor (impala: *n* = 150; kudu: *n* = 64). Because LST and year were strongly correlated, we decided not to include year in the model (Nakagawa and Cuthill, 2007).

## Results

### Impala

The model selection revealed that best-fitting model to explain the variation in impala FGM concentrations included an interaction between management area and temperature and was significantly better than the next best model (∆AICc > 2; Table 1).

**Table 1:** Model comparisons among five proposed models explaining the variation in FGM concentrations in Ruaha–Rungwa ecosystem for both Impala and Kudu.

**Impala**	**Model**	**Structure**	**df**	**∆AICc**	**Weight**
	LST	log(FGM) ~ Area ^*^ LST^2^ + NDVI + Group size + (1|groupID)	10	0.00	0.99
	NDVI	log(FGM) ~ Area ^*^ NDVI + LST^2^ + Group size + (1|groupID)	9	13.96	0.01
	No interaction	log(FGM) ~ Area + NDVI + LST^2^ + Group size + (1|groupID)	8	17.82	0.00
	Group size	log(FGM) ~ Area ^*^ Group size + LST^2^ + NDVI + (1|groupID)	9	24.96	0.00
	*(null)*	log(FGM) ~ 1 + (1|groupID)	3	48.64	0.00
**Kudu**	LST	log(FGM) ~ Area ^*^ LST^2^ + NDVI + Group size + (1|groupID)	10	0.00	0.87
	NDVI	log(FGM) ~ Area ^*^ NDVI + LST^2^ + Group size + (1|groupID)	9	4.16	0.11
	No interaction	log(FGM) ~ Area + NDVI + LST^2^ + Group size + (1|groupID)	8	7.82	0.02
	Group size	log(FGM) ~ Area ^*^ Group size + LST^2^ + NDVI + (1|groupID)	9	14.43	0.00
	*(null)*	log(FGM) ~ 1 + (1|groupID)	3	17.77	0.00

Fixed effects include of Area (RGR vs. RNP), NDVI = Normalized Difference Vegetation Index, LST = mean daily land surface temperature and group size. Non-independence within animal group was accounted for adding group identity (groupID) as a random factor. Degrees of freedom (df) are given, as well as difference in Akaike’s Information Criterion adjusted for small samples sizes (∆AICc) and their respective weights

**Table 2:** Model estimates from the linear mixed-effects model for impala explaining the variation in FGM concentrations in Ruaha–Rungwa ecosystem.

**Fixed effects**	**Estimate**	**SE**	**df**	***t***	***P***	
*(Intercept)*	7.27	0.51	131.23	14.16	< 0.001	***
Area: RGR	−0.75	0.21	137.27	−3.63	< 0.001	***
LST (lin.)	−5.88	2.76	149.84	−2.13	0.035	*
LST (qua.)	−8.28	2.76	139.17	−3.00	0.003	**
NDVI	−3.27	1.55	129.36	−2.11	0.037	*
Group size	−0.01	0.00	156.55	−3.60	< 0.001	***
Area: LST (lin.)	0.10	3.14	155.87	0.03	0.974	
Area: LST (qua.)	11.36	3.11	146.41	3.65	< 0.001	***
**Random effects**	**Variance**	**SD**				
Group ID	0.511	0.715				
Residual	0.419	0.647				

Significance codes: *P* < 0.001 ^***^; 0.001–0.01 ^**^; 0.01–0.05 ^*^; 0.05–0.1.

Model estimates for the effects of Area (RGR vs. RNP), NDVI = Normalized Difference Vegetation Index, LST = mean daily land surface temperature and group size. Non-independence within animal group was accounted for adding group identity as a random factor

**Figure 2:**
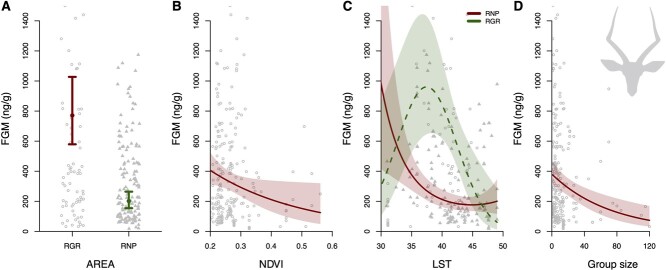
Changes in average FGM concentrations due to management regime and environmental factors in Common Impala in Ruaha–Rungwa ecosystem. The effect of (A) management regime (RGR, hunted, red solid lines/open circles vs. RNP, non-hunted, green dashed lines/solid triangles), (B) NDVI, (C) mean daily LST in interaction with management regime and (D) group size, on impala FGM concentrations. Model estimates are represented as points or lines; 95% confidence intervals are the arrows or the shaded areas. The Y-axis is truncated at 1500 ng/g to aid in the interpretation of the results.

We found that FGM concentrations were significantly lower in the non-hunting area (RNP) when compared to the areas where trophy hunting is allowed (RGR); FGM levels in RNP (mean ± SE = 202 ± 28 ng/g) were 74% lower compared to RGR (mean ± SE = 771 ± 112 ng/g; Table 2; Fig. 2). NDVI had a significant negative effect on FGM levels, such that impala FGM levels decreased 73% from 406 ng/g (95% Confidence interval [CI] = 314–524 ng/g) at low NDVI levels (NDVI = 0.2) to 110 ng/g (95% CI = 38–317 ng/g) at high NDVI levels (NDVI = 0.6). LST had an overall significant negative effect on FGM levels, such that impala FGM levels decreased 80% from 677 ng/g (95% CI = 295–1553 ng/g) at low LST levels (LST = 29) to 133 ng/g (95% CI = 70–252 ng/g) at high LST levels (LST = 49). However, for impala in RNP, FGM levels were highest (mean [95% CI] = 1247 ng/g [445–3494 ng/g]) at lowest LST (LST = 29) and declined to their lowest value (mean [95% CI] = 177 ng/g [137–229 ng/g]) at an LST of 44, and increase marginally with further increases of LST. Impala in RGR showed a strong quadratic relationship, with lowest FGM levels at lowest (mean [95% CI] = 217 ng/g [61–766 ng/g]) and highest LST (mean [95% CI] = 61 ng/g [13–288 ng/g]), and highest FGM levels at mean LST of 39 (mean [95% CI] = 916 ng/g [648–1295 ng/g]; Fig. 2). Lastly, group size had a significant negative effect on FGM levels, such that impala FGM levels decreased 74% from 375 ng/g (95% CI = 305–462 ng/g) at small group sizes (group size = 1) to 97 ng/g (95% CI = 49–192 ng/g) at large group sizes (group size = 100).

**Figure 3:**
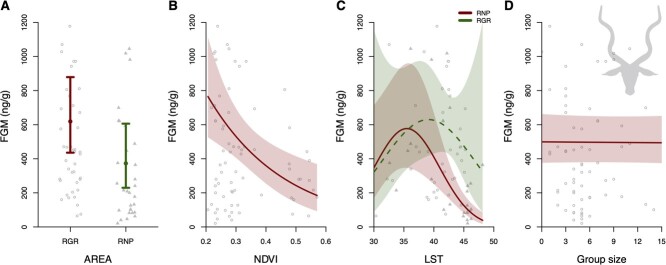
Changes in average FGM concentrations due to management regime and environmental factors in Greater Kudu in Ruaha–Rungwa ecosystem. The effect of (A) management regime (RGR, hunted, red solid lines/open circles vs. RNP, non-hunted, green dashed lines/solid triangles), (B) NDVI, (C) mean daily LST in interaction with management regime and (D) group size (x-axis is truncated at 15, as model is not informative on larger group sizes due to a lack of data), on kudu FGM concentrations. Model estimates are represented as points or lines; 95% confidence intervals are the arrows or the shaded areas. The Y-axis is truncated at 1300 ng/g to aid in the interpretation of the results.

The model explained 67% of the variation in FGM levels in our dataset; the fixed effects alone explained 26% of the variation in FGM levels.

### Greater kudu

Similarly, the best-fitting model to explain the variation in kudu FGM concentrations included an interaction between management area and temperature and was significantly better than the next best model (∆AICc > 2; Table 1).

Kudu FGM levels were significantly lower in RNP when compared RGR; FGM levels in RNP (mean ± SE = 373 ± 90 ng/g) were 40% lower compared to RGR (mean ± SE = 619 ± 109 ng/g; Supplementary Table S1; Fig. 3A). NDVI had a significant negative effect on FGM levels (Supplementary Table S1), such that kudu FGM levels decreased 79% from 785 ng/g (95% CI = 533–1155 ng/g) at low NDVI levels (NDVI = 0.2) to 165 ng/g (95% CI = 77–352 ng/g) at high NDVI levels (NDVI = 0.6; Fig. 3B). LST did not have an overall significant effect on FGM levels. There was insufficient data for kudu in RGR, indicated by the large error bars in Figure 3C. However, for kudu residing in RNP, there was a small but significant decrease in FGM levels from highest FGM levels at an LST of 35 (mean [95% CI] = 577 ng/g [437–960 ng/g]; Fig. 3C) to lowest FGM levels at highest LST (LST = 48; mean [95% CI] = 39 ng/g [18–86 ng/g]). Lastly, in contrast to impala, group size did not have a significant effect on FGM levels (Supplementary Table S1; Fig. 3D).

**Figure 4:**
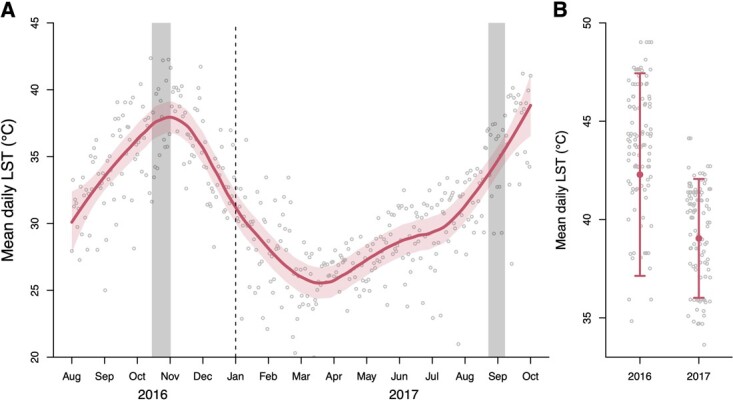
Mean daily LST change over time in Ruaha–Rungwa ecosystem during 2016–2017. LST varied greatly throughout the seasons (A) and between years (B). Sampled individuals experienced significantly higher LST in 2016 as compared to 2017 (B). In (A) daily LST values (gray circles) and the daily average LST (red line) for the entire study area, from August 2016 to October 2017 are shown, as well as the timing of the two study period (shaded gray area). Note that 95% confidence interval is the shaded red area or the arrows.

The model explained 50% of the variation in FGM levels in our dataset; the fixed effects alone explained 39% of the variation in FGM levels.

## Discussion

We found that FGM levels—a measure of physiological stress—were higher in both impala and greater kudu in RGR, where trophy hunting is allowed, compared to animals residing RNP indicating the potential negative impacts of trophy hunting on the wildlife stress response. We also show that both the environmental and social context considerably affected an animal’s stress response. Both ungulates responded very similar to anthropogenic and environmental disturbances, though only impala had a lower stress response with increasing group size.

### Effect of hunting on FGM levels

Our results show that both impala and kudu FGM levels were significantly higher in RGR populations compared to RNP populations. We hypothesized that FGM levels would be higher in RGR, where wildlife trophy hunting is normally conducted as the main tourism activity, and lower in RNP, where photographic tourism is the main tourism activity. The trophy hunting season spans from September to October, the same period of sample collection—there were 63 trophy hunters in 2016 and 39 trophy hunters in 2017 in RGR (pers. comm.: Tourism Officers in charge at RGR). The observed difference might be due to the nature of trophy hunting, which is likely to induce a higher disturbance to ungulate populations in RGR. Several studies have shown that animals in highly disturbed areas (e.g. human settlements and intensive livestock grazing) had higher levels of FGM than in protected areas where such activities are not allowed (Rehnus *et al.,* 2014).We assumed that populations in both study areas were subject to similar levels of illegal hunting, habitat loss, predation pressure, and human–wildlife conflict (HWC) as both areas form a single continuous ecosystem (Fig. 1). Therefore, the main difference between the two areas in terms of potential disturbances should therefore be trophy hunting in the RGR.

### Effect of forage availability on FGM levels

We found that both impala and kudu FGM levels decreased sharply with increasing forage availability (measured as NDVI). Multiple studies have used NDVI as a proxy for forage availability or dietary protein and used this proxy to relate forage availability to FGM concentrations in wildlife (Stabach *et al.,* 2015; Oduor *et al.,* 2020; Hunninck *et al.,* 2020b). For instance, Stabach *et al.* (2015) observed a similar negative relationship between FGM concentrations and NDVI in wildebeest (*Connochaetes taurinus*) in Kenya. Pokharel *et al.* (2019) also demonstrated this effect between FGM concentrations and NDVI in free-ranging Asian elephants (*Elephas maximus*) in India, while Hunninck *et al.* (2020b) found strong evidence of this negative relationship between FGM concentrations and NDVI in impala in the Serengeti ecosystem. NDVI represents the greenness of a landscape of both woody and non-woody plants, and although the variation in NDVI is mainly due to variation in grassy vegetation, new growth on woody vegetation—which is more nutritious than mature leaves—also affects NDVI considerably (Pettorelli *et al.,* 2011; Hunninck *et al.,* 2020b). Therefore, while impala are grazers and kudu prefer to browse, high NDVI is likely to correlate to better forage quality for both species. GCs have an important role in an animal’s energy balance, and a decrease in energy uptake has been shown to results in an increase in energy mobilization from other sources, through an upregulation of GCs (Strack *et al.,* 1995). Our findings therefore corroborate previous studies, indicating that a decrease in vegetation quality can have a potent positive effect on the adrenocortical response in wildlife (Hunninck *et al.,* 2020b).

### Effect of temperature on FGM levels

Impala experienced lowest FGM levels at mean temperatures (i.e. LST) in RNP (where hunting is prohibited) while FGM levels increased toward lowest and highest temperatures. We expected higher levels of FGM at lowest and highest temperatures because animals should increase energy mobilization to facilitate more increased physiological thermoregulation. Interestingly, impala FGM levels were more than twice as high at mean temperatures in RGR (where hunting is allowed) compared to the RNP populations; FGM levels at high temperatures were similar in both areas (Fig. 2C). Impala are sensitive to heat fluctuations and have been shown to experience heat stress above 35°C and extreme heat can negatively affect their diurnal activity (Maloiy and Hopcraft, 1971; Shrestha *et al.,* 2014). Although the data for kudu in RGR were inconclusive, a similar but weak negative relationship between FGM levels and temperature was observed in kudu in RNP. Higher FGM concentrations at lower temperatures might be a result of higher diurnal activity, which has been shown to correlate with FGMs. Higher diurnal activity could also increase encounter rate between impala and anthropogenic stressors, further contributing to elevated FGM levels, especially in RGR, where hunting is allowed. Both negative (Corlatti *et al.,* 2011; Santos *et al.,* 2018) and positive relationships (Millspaugh *et al.,* 2001) between FGM and ambient temperatures have been observed in ungulates before. More studies are necessary to disentangle the exact mechanisms GCs facilitate thermoregulation in both cold and hot environments in wild tropical ungulates.

### Effect of group size on FGM levels

FGMs levels were higher in impala in smaller groups compared to impala in larger groups. This finding supports our hypothesis that FGMs levels would be higher in small groups (impala group sized ranged between 1 and 120 individuals). An animal’s social environment can have important effects their stress response (Creel *et al.,* 2013). Larger groups benefit from increased group vigilance, while individual level vigilance can be reduced, thereby freeing up time and energy for foraging, that is, many-eyes hypothesis (Pulliam, 1973). Combined with a reduced predation risk (dilution effect; Hamilton, 1971) in large groups, larger group size may have a buffering effect of the stress response, decreasing FGM levels (Hennessy *et al.,* 2009).

Kudu did not have a significant effect of group size on FGM levels (Supplementary Table S1). This might be largely due to the relatively small group sizes encountered in our study population: only 4 out of 64 groups consisted of more than 14 individuals, while the median group size for kudu was only 5 individuals. More data from larger groups would be needed to establish if larger group sizes affect individual FGM levels in kudu.

## Conclusion

Our results show that two common ungulates in Ruaha–Rungwa ecosystem were subject to both anthropogenic and environmental disturbances, which significantly elevated their GC concentrations. FGM levels were substantially lower in a strictly protected area, where hunting is not allowed, indicating that the sublethal effects of hunting could strongly affect animal populations. Furthermore, lower forage availability and higher ambient temperatures also elevated physiological stress levels, suggesting that with increasingly severe climate variability, herbivore populations could also suffer secondary physiological effects. These results highlight the importance of protected areas and their role to minimize chronic physiological stress in animals. Our findings therefore emphasize the importance of national parks in maintaining healthy ungulate populations.

## Funding

We acknowledge the EU-funded project *AfricanBioServices* (grant number 641819) that assisted us with field grants.

## Author contributions

K.M.H., R.D.F. and E.R. designed the study. K.M.H. and the field assistants collected fecal samples. K.M.H. and TAWIRI colleagues extracted fecal samples, while they were being analyzed in RPs lab. L.H. conducted the statistical analyses. K.M.H., L.H. and P.S.R. lead the writing of the manuscript. E.R., K.M.H. and R.D.F. acquired funding. All authors contributed significantly to writing and editing the manuscript and with helpful discussions. Two anonymous reviewers greatly helped in improving this manuscript.

## Data availability statement

The data that support the findings of this study are available from the corresponding author upon reasonable request.

## Supplementary Material

Web_Material_coad002

## Supplementary material

The following file is available at Conservation Physiology online: Supplementary Table S1: Model estimates from the linear mixed-effects model for Greater kudu explaining the variation in FGM concentrations in Ruaha–Rungwa ecosystem.
