# Supplementary material for: Between hunter and climate: the effects of hunting and environmental change on fecal glucocorticoid metabolite levels in two sympatric ungulate species in the Ruaha–Rungwa ecosystem, Tanzania
Source: Conserv Physiol. 2023 Feb 7;11(1):coad002. doi: 10.1093/conphys/coad002 (PMC10660377; doi:10.1093/conphys/coad002)
Supplement: Web_Material_coad002 [file web_material_coad002.docx]

**Supplementary information**

**Table S1:** Model estimates from the linear mixed-effects model for Greater kudu explaining the variation in fecal glucocorticoid metabolite (FGM) concentrations in Rungwa-Ruaha Ecosystem. Model estimates for the effects of Area (RGR = Rungwa Game Reserve vs. RNP = Ruaha National Park), NDVI = Normalized Difference Vegetation Index, LST = mean daily Land Surface Temperature, and Group size. Non-independence within animal group was accounted for adding group identity as a random factor.

| **Fixed effects** | **Estimate** | **SE** | **df** | **t-value** | **p-value** |  |
| --- | --- | --- | --- | --- | --- | --- |
| *(Intercept)* | 7.50 | 0.47 | 66.48 | 15.816 | < 0.001 | *** |
| Area - RGR | -0.69 | 0.26 | 52.57 | -2.643 | 0.011 | * |
| LST (lin.) | 0.39 | 1.53 | 65.75 | 0.253 | 0.801 |  |
| LST (qua.) | -1.78 | 1.77 | 62.96 | -1.007 | 0.318 |  |
| NDVI | -3.90 | 1.24 | 66.51 | -3.151 | 0.002 | ** |
| Group size | 0.00 | 0.01 | 68.57 | -0.132 | 0.895 |  |
| Area : LST (lin.) | -4.63 | 1.88 | 56.96 | -2.468 | 0.017 | * |
| Area : LST (qua.) | -1.91 | 2.09 | 55.99 | -0.912 | 0.366 |  |
| **Random effects** | **Variance** | **SD** |  |  |  |  |
| Group ID | 0.127 | 0.356 |  |  |  |  |
| Residual | 0.568 | 0.754 |  |  |  |  |
| \| *Significance codes: p < 0.001 ***; 0.001 - 0.01 **; 0.01 - 0.05 *; 0.05 - 0.1.* \| \| --- \| | | | | | | |
